# A CITATION REVIEW OF DISSEMINATION AND IMPLEMENTATION MODELS UTILIZED IN AGING RESEARCH WITHIN THE UNITED STATES

**DOI:** 10.1093/geroni/igac059.1537

**Published:** 2022-12-20

**Authors:** Jennifer Sullivan, Anna Rae Montano, Heather Davila, Marlena Shin, Chelsea Hawley, Jaime Hughes, Kelly O'Malley, Camilla Pimentel

**Affiliations:** VA LTSS COIN / Brown University, Providence, Rhode Island, United States; VA Providence Healthcare System, Providence, Rhode Island, United States; VA Iowa, Iowa City, Iowa, United States; VA Boston Healthcare System, Boston, Massachusetts, United States; VA Bedford Healthcare System, Bedford, Massachusetts, United States; Wake Forest, Wake Forest, North Carolina, United States; VA Boston Healthcare System, Boston, Massachusetts, United States; VA Bedford Healthcare System, Bedford, Massachusetts, United States

## Abstract

The application of implementation science in aging research has been growing. To our knowledge, there has been no study detailing the Dissemination and Implementation (D&I) Models utilized in aging research. The goal of this citation review is to further understand D&I models frequency and nature of their use in aging research. We identified 111 Dissemination and Implementation (D&I) Models compiled on the dissemination-implementation.org website. We then conducted a citation analysis on them, searching Web of Science and PubMed databases. We extracted key data from identified articles up to January 28, 2022. Search terms were broad and included aging, older, elderly and geriatric. To be included, articles had to be in peer-reviewed journals, in English, and occur in the United States. We identified 297 articles meeting our eligibility criteria. The nature of the way D&I models used to advance evidence-based practice in aging research and practice varied as did the number of citations over time. Of the D&I models included in this review, only one (4E Framework) was developed within the aging research field. The top five models included: CFIR, RE-AIM 1.0, Behavior Change Wheel, Greenhalgh Diffusion of innovation in Service Organization and CBPR. Citations were distributed across many frameworks and yet only totaled less than 1% of all D&I Model citations suggesting there are many ways the field can grow in the future.

